# The Role of IL-33 in Experimental Heart Transplantation

**DOI:** 10.1155/2020/6108362

**Published:** 2020-03-19

**Authors:** Jie Chen, Yan He, Zhongming Xie, Yingying Wei, Lihua Duan

**Affiliations:** ^1^Department of Scientific Research and Education, Jiangxi Provincial People's Hospital Affiliated to Nanchang University, Nanchang, Jiangxi, China; ^2^Department of Rheumatology and Clinical Immunology, The First Affiliated Hospital of Xiamen University, Xiamen, Fujian, China; ^3^Department of Rheumatology and Clinical Immunology, Jiangxi Provincial People's Hospital Affiliated to Nanchang University, Nanchang, Jiangxi, China

## Abstract

Interleukin-33 (IL-33) is a member of the IL-1 family of proteins that are produced by a variety of cell types in multiple tissues. Under conditions of cell injury or death, IL-33 is passively released from the nucleus and acts as an “alarmin” upon binding to its specific receptor ST2, which leads to proinflammatory or anti-inflammatory effects depending on the pathological environment. To date, numerous studies have investigated the roles of IL-33 in human and murine models of diseases of the nervous system, digestive system, pulmonary system, as well as other organs and systems, including solid organ transplantation. With graft rejection and ischemia-reperfusion injury being the most common causes of grafted organ failure or dysfunction, researchers have begun to investigate the role of IL-33 in the immune-related mechanisms of graft tolerance and rejection using heart transplantation models. In the present review, we summarize the identified roles of IL-33 as well as the corresponding mechanisms by which IL-33 acts within the progression of graft rejection after heart transplantation in animal models.

## 1. Experimental Heart Transplantation

In the field of heart transplantation in recent decades, much progress has been made in elucidating the mechanisms of cardiac graft rejection. Allograft rejection is now considered one of the most common causes of graft failure after cardiac transplantation [1, 2]. Currently, both acute and chronic rejection following heart transplantation are generally believed to result from a T helper 1 (Th1) cell-dominated immune response, which is characterized by the massive production of several certain types of proinflammatory cytokines, including tumor necrosis factor-*α* (TNF-*α*), interleukin-2 (IL-2), and interferon-*γ* (IFN-*γ*) [3–5]. These cytokines have multiple effects on immune cells and the immune response, and their overproduction promotes graft destruction and dysfunction by mechanisms such as inducing the expression of costimulatory molecules, major histocompatibility complex- (MHC-) II, and chemokines in the graft; facilitating the induction of alloantigen-specific cytotoxicity; activating graft-infiltrating macrophages and macrophage-mediated effector mechanisms; and inducing alloantibody class switching to complement-fixing immunoglobulin G (IgG)2a [6–12]. Moreover, transplant-induced alloreactive Th1 cells enhance delayed-type hypersensitivity and activate B cells to produce alloreactive antibodies [13]. On the contrary, the Th2 type response and type 2 cytokines, for example, IL-4 and IL-5, have been implicated in graft tolerance during the progress of allograft rejection [14, 15]. Studies involving the adoptive transfer of Th cell lines revealed different effects of Th1 and Th2 cells in transplant rejection. Notably, rejection was slightly delayed in mice treated with Th2 cells compared with that in mice treated with Th1 cells [16, 17]. Thus, to prolong allograft survival and maintain the physiological functioning of the graft, a shift in the posttransplantation Th1/Th2 balance in response to donor-derived signals is particularly vital to prolonging allograft survival. However, it must be noted that Th2 cytokines also have been found to activate eosinophils, which are thought to mediate allograft rejection [18].

Recently, following the discovery of a new T-cell subset, Th17, investigations into the correlation between Th17 cells (or the corresponding specific cytokine, IL-17) and allograft rejection, especially cardiac allograft rejection, have been reported with the results revealing the detrimental roles of Th17 cells and IL-17 [19–22]. Moreover, regulatory T cells (Tregs) have been found to play pivotal roles in the induction of allograft tolerance. To date, studies of Tregs in transplantation have focused on non-antigen-specific thymus-derived naïve CD4+CD25+FOXP3+ T cells, which are fresh or activated by IL-2 only or IL-2 plus anti-CD3 antibody [23, 24]. These cells express the transcription factor FOXP3, which inhibits IL-2 transcription, and promote the induction of transplant tolerance [25, 26], typically via cell contact-dependent and cell contact-independent mechanisms, ranging from cytokine release, receptor endocytosis, and purinergic signaling to cell cytotoxic mechanisms [27–29]. Thus, they have a wide range of inhibitory effects on immune responses, including inhibition of the proliferation and activation of CD4+ and CD8+ T cells, suppression of B-cell responses, and regulation of macrophage and natural killer cell functions [30].

In agreement with these observations, additional studies have reported that the balances of Tregs/Th17 and Th1/Th2 are crucial for allograft survival [31–33]. Moreover, it has been reported that both Th1 and Th2 cytokines, including IFN-*γ* and IL-5, have the ability to promote the survival of alloantigen-specific CD4+T regulatory cells [34, 35]. However, another typical Th2 cytokine, IL-4, has been found to not maintain alloantigen-specific CD4+CD25+ Tregs [36]. These observations revealed interactions between Tregs and Th cells, which indicated that the corresponding mechanisms are rather complicated.

Unfortunately, the desperate shortage of acceptable donors for transplantation persists, particularly for cardiac donors [37], and concordant organ xenotransplantation has been considered a new solution based on several studies that have emerged recently. However, only a few studies have focused on the underlying mechanisms of immune rejection following xenotransplantation. The mouse-to-rat, rat-to-mouse, hamster‐to‐rat, monkey-to-baboon, pig-to-monkey, and pig-to baboon experimental xenotransplantation models have been applied in these studies to investigate the rejection mechanisms for concordant cardiac xenotransplantation [38–51]. Concordant xenotransplantation can almost overcome acute vascular rejection due to differences between species, and with the use of certain types of immunosuppressive agents or treatments, xenograft survival can be extended from several days to as long as 300 days [40, 42, 43, 45, 47]. Despite these promising results, the underlying mechanisms of rejection remain to be demonstrated.

## 2. Overview of IL-33

Since its identification as a member of the IL-1 family in 2005 [52], IL-33 has been found to play key roles in both innate and adaptive immunity [53]. IL-33 is constitutively expressed in the nuclei of various cell types in humans and mice in steady state, including epithelial, endothelial, and fibroblast-like cells [54, 55]. Notably, when these cells are destroyed (cell death by injury, necrosis, or apoptosis) and the intact membranes are breached, the IL-33 “stored” in the nucleus is passively released [56]. Accordingly, IL-33 has been termed an alarmin or damage-associated molecular pattern (DAMP), similarly to high mobility group box 1 (HMGB1) and IL-1*α* [57].

The released bioactive IL-33 initiates its downstream signaling by binding to its specific receptor ST2. Two isoforms of ST2 are produced via alternative splicing, the soluble form (sST2) and membrane-bound form (ST2) [58]. sST2 is a decoy receptor for IL-33 that can bind IL-33 without initiating the intracellular signaling [59]. Transmembrane ST2, also designated ST2L, was first found to be selectively and stably expressed by Th2 cells, and upon binding to IL-33, ST2L mediates Th2 cell functions such as the expression of the cytokines IL-4, IL-5, and IL-13 [9, 52, 60]. In addition, resident immune cells, including mast cells, Tregs, and group 2 innate lymphoid cells (ILC2s), were also found to constitutively express ST2L [61–69]. IL-33 acts through these major target cells by binding to ST2L on the cell surface and is intensively involved in various diseases. Examples of IL-33 activity include the exacerbation of experimental autoimmune liver injury [70], amelioration of experimental inflammatory bowel disease [71], and induction of allograft tolerance [9, 72, 73].

## 3. IL-33 and Allograft Ischemia-Reperfusion (IR) Injury

As an “alarmin” that is released from cell nuclei following cell death by necrosis or apoptosis, IL-33 has been shown to induce protective effects in neighboring cells [74, 75]. During the process of solid organ transplantation, the cold preservation of organs and reperfusion afterwards is of central importance in the success of the transplantation and cell death can easily occur during this process [76]. Research has shown that IR injury is often closely related to an increased incidence of cardiac graft rejections [77]. However, additional studies have reported a protective role of IL-33 in IR injury of solid organs [78–81]. Moreover, the beneficial effects of IL-33 released in the process of cardiac IR and IR-induced myocardial injury have also been demonstrated [82–84]. IL-33 expression at both the mRNA and protein levels was found to be increased during myocardial IR [84]. Encouragingly, after IR injury, IL-33 treatment significantly reduced the myocardial infarct size and the expression of biomarkers of myocardial damage including cardiac troponin I (cTnI), lactate dehydrogenase (LDH), and creatine kinase (CK); markedly inhibited I/R-induced apoptosis of myocardiocytes; and reduced the inflammatory response in myocardial I/R by decreasing the expression of the proinflammatory cytokine HMGB1, which plays a deleterious role in myocardial IR [75, 85, 86] and upregulates the expression of classic Th1 proinflammatory cytokines (tumor necrosis factor-*α* (TNF-*α*) and IL-6) [75, 84]. As further confirmation of these effects of IL-33 and ST2L binding, the anti-inflammatory and antiapoptotic effects of IL-33 were found to be suppressed in ST2(-/-) mice [83] or upon inhibition of the p38 MAPK signaling pathway, which is a known IL-33/ST2 downstream signaling pathway in IR injury [84, 87]. Based on evidence that IL-33 activates the p38 MAPK signaling pathway to inhibit TNF-*α* and IL-6 expression in the myocardium [88], that in the context of liver IR injury, IL-33 upregulates the expression of antiapoptotic proteins by activating the p38 MAPK signaling pathway [78], and that the p38 MAPK signaling pathway is involved in HMGB1 release [89, 90], it can be presumed that in heart IR injury, IL-33 activates p38 MAPK signaling to inhibit the release of HMGB1 and then leads to downstream anti-inflammatory effects including the decreased production of cytokines such as TNF-*α* and IL-6.

## 4. IL-33 and Cardiac Allograft Transplantation

Acute and chronic rejection caused by an immune response towards alloantigens is a major and serious limitation in the clinical success of cardiac allograft transplantation. In clinical trials, sST2, the decoy receptor of IL-33, was reported to be a marker for acute rejection after cardiac allotransplantation, as its serum level was found to elevate during acute rejection, compared to the prerejection period, and to decrease again after treatment for acute rejection [91]. Moreover, studies of experimental cardiac allograft transplantation in a mouse heterotopic heart transplantation model have reported the therapeutic capacity of IL-33 for inhibiting the progression of acute and chronic rejection and prolonging graft survival [9, 72, 73]. Among those studies, Yin et al. were the first to report the beneficial effects of IL-33 for promoting cardiac allograft survival [9]. They specifically investigated the ability of IL-33 to shift the type of T-cell response during the process of alloreaction. They first found that graft survival was significantly extended (from 7.2 ± 1.2 days to 21.7 ± 1.6 days) with the administration of exogenous recombinant IL-33 to the recipient mice daily from before the day of transplantation to day 7 after surgery [9]. Then, based on the demonstration of ST2L expression on Th2 cells but not Th1 cells, they further observed that IL-33 treatment in vitro induced the production of IL-5 and IL-13 in Th2 cells, while also decreasing IFN-*γ* production by Th1 cells [9]. In vivo tests showed similar effects on the mRNA and protein levels of cytokines, with recipient splenic IL-4 (a prototypic Th2 cytokine) expression being obviously increased and IFN-*γ* (a prototypic Th1 cytokine) expression being reduced upon IL-33 treatment [9]. Moreover, along with IL-4 upregulation, recipient mice treated with IL-33 had greater IgG1 and IgM concentrations in the sera but lower IgG2a expression [9]. Thus, it was concluded that IL-33 can facilitate cardiac allograft tolerance by shifting the Th1/Th2 balance to promote Th2 immune deviation and Th2-polarized naïve T-cell cytokine production [9].

A study by Turnquist et al. later demonstrated the capacity of IL-33 to induce the generation of suppressive cell groups in cardiac allograft rejection [72]. They also confirmed the prolongation of graft survival with IL-33 treatment based on the observation that IL-33 delivery into the recipients tripled the graft survival time (mean survival time of 29 days versus 9 days in control groups) [72]. In further experiments, they found that the beneficial effects of IL-33 treatment are based on the expansion of several suppressive or regulatory cell groups, including CD4+Foxp3+ Tregs, and especially ST2+ Tregs, poorly stimulatory CD11b+ cells, and more importantly, CD11b+Gr-1^int^ myeloid-derived suppressor cells (MDSCs) [72]. MDSCs are a cell group consisting of immature myeloid cells and myeloid progenitor cells that has the potent ability to suppress T-cell responses [92–94]. Moreover, they also found that a single dose of IL-33 therapy reduced serum IL-12p40/p70 expression and increased the circulating levels of IL-5 and IL-13 [17], which partially agree with the in vitro observations of Yin et al. Notably, experiments in ST2-/- mice demonstrated consistent results and confirmed that the therapeutic benefit of IL-33 is dependent on the recipient expression of ST2 [72]. Thus, based on the collective results of these studies, it can be concluded that IL-33 possesses immunoregulatory properties in acute allograft rejection by supporting type 2 T-cell responses and expanding immunosuppressive cell groups including MDSCs and Tregs.

While the above studies focused on acute cardiac allograft rejection, Brunner et al. investigated the impact of IL-33 on the chronic response using a chronic cardiac rejection model [73]. Similarly, they found that IL-33 had beneficial effects on prolonging allograft survival during chronic cardiac rejection [73], and their studies revealed that the protective effects of IL-33 were based on multiple mechanisms. These included the capacity of IL-33 to induce the accumulation of immunosuppressive Tregs and MDSCs in the spleen and within the graft; to increase the production of IL-5, IL-10, and IL-13; to reduce the number of B220+CD19+B cells as well as alloantibody expression; and to decrease production of the proinflammatory cytokine IL-17A [73]. All these observations proved the benefit of IL-33 therapy in ameliorating chronic cardiac rejection, which is consistent with the conclusion that IL-33 has protective effects against the acute alloresponse.

It is worth mentioning that sST2, the decoy receptor of IL-33, has been found to play a role in the progression of clinical heart transplantation rejection. First, based on clinical observations, Pascual-Figal et al. identified sST2 as a marker of acute cardiac allograft rejection in patients, as the concentrations of sST2 varied according the presence of acute rejection and showed a predictive ability, when considered in combination with N-terminal pro-B-type natriuretic peptide (NT-proBNP) expression, for biochemical identification of rejection [91]. Following that report, a similar investigation drew an opposite conclusion that sST2 has limited ability to predict acute allograft rejection in heart transplantation patients [95], but more recent studies have confirmed that elevated serum levels of sST2 correlate with incidence of pediatric heart transplantation rejection [96] and increased risk for antibody-mediated alloreaction [97]. Therefore, these observations supporting the relationship between sST2 and acute heart transplantation rejection provide further evidence of the role of IL-33 signaling in allograft transplantation.

## 5. IL-33 and Concordant Cardiac Xenotransplantation

Acute humoral xenograft rejection (AHXR) occurs within 3 days after transplantation in the mouse-to-rat and hamster-to-rat heart transplantation models. Previous studies have shown that IL-33 plays a deleterious role associated with the activation and production of Th2 type cytokines and alternatively activated macrophage (AAM) polarization in many diseases [13, 14]. To date, very few studies have investigated the protective or deleterious roles of IL-33 in concordant transplantation. One study using a mouse-to-rat cardiac xenotransplantation model [43] found that treatment with a combination of IL-33 and half-dose leflunomide (Lef) prolonged the survival of the xenograft, indicating that IL-33 may also have protective effects on xenografts. Further research revealed the underlying tolerogenic mechanisms which involved the inhibition of T-cell proliferation, the reduction of Th1 type cytokine IFN-*γ* production, and an increase in the number of CD4+Foxp3+ Tregs in the recipient response [43]. It is worth noting that the administration of IL-33 alone could not induce effective tolerance of the xenograft; this effect was only achieved with the combination of IL-33 and Lef, which differs from the results obtained in allograft models [43]. Nevertheless, the research indicates that IL-33 may also play a beneficial role in protecting against xenograft rejection.

In contrast to its effects on allograft transplantation, IL-33 treatment alone has no effect on xenograft survival. Thus, at present, AHXR can only be suppressed with treatment with a B-lymphocyte inhibitor.

## 6. Conclusions and Perspectives

This minireview summarizes the functions of IL-33 as well as the underlying molecular mechanisms in protecting against heart allograft and xenograft rejection. Briefly, IL-33 is originally stored in nucleus and passively released by necrotic or apoptotic cells upon IR injury during the early stage after transplantation. The released IL-33 binds to its receptor ST2 on Th2 cells, leading to increased production of Th2 type cytokines and an upset in the balance of Th1/Th2 responses. Th1 cell functions and production of Th1 type cytokines are inhibited, whereas Th2 responses are enhanced. The result is the amelioration of graft rejection. Meanwhile, suppressive cell groups including MDSCs and Tregs expressing ST2 are induced by IL-33 release, further facilitating the tolerogenic state to the graft. Overall, IL-33 possesses beneficial effects throughout the complete process of heart transplantation by reducing IR injury and ameliorating graft rejection.

Although the presented data suggest that IL-33 is a promising target for the prevention and intervention of cardiac graft rejection, the underlying mechanisms especially the signaling pathways deserve further investigation. With regard to the clinical implications, the research to date indicates that combined use of IL-33 and an immunosuppressant may achieve better therapeutic tolerant effects than the use of IL-33 alone. Furthermore, for possible clinical xenotransplantation in the future, IL-33 may also have protective effects via the inhibition of the immune response against the xenograft. However, as IL-33 has multiple effects in a wide range of tissues and cells and promotes the Th2 cell response, the potential for IL-33 therapy to aggravate Th2-induced diseases has not been clearly studied yet. Before IL-33 can be applied clinically to promote transplant tolerance and prolong graft survival, this possibility must be deeply investigated.
